# The effect of relative hypotension on 30-day mortality in older people receiving emergency care

**DOI:** 10.1007/s11739-023-03468-5

**Published:** 2023-11-08

**Authors:** James David van Oppen, Rhiannon Kate Owen, William Jones, Lucy Beishon, Timothy John Coats

**Affiliations:** 1 Department of Population Health Sciences, University of Leicester, Leicester, UK; 2 Emergency and Specialist Medicine, University Hospitals of Leicester NHS Trust, Leicester, UK; 3 Swansea University Medical School, Swansea University, Swansea, UK; 4 Department of Cardiovascular Sciences, University of Leicester, Leicester, UK; 5 NIHR Leicester Biomedical Research Centre, British Heart Foundation Cardiovascular Research Centre, Glenfield Hospital, Leicester, UK

**Keywords:** Emergency care, Geriatrics, Early warning score, Physiology

## Abstract

Research has observed increased mortality among older people attending the emergency department (ED) who had systolic pressure > 7 mmHg lower than baseline primary care values. This study aimed to (1) assess feasibility of identifying this ‘relative hypotension’ using readily available ED data, (2) externally validate the 7 mmHg threshold, and (3) refine a threshold for clinically important relative hypotension. A single-centre retrospective cohort study linked year 2019 data for ED attendances by people aged over 64 to hospital discharge vital signs within the previous 18 months. Frailty and comorbidity scores were calculated. Previous discharge (‘baseline’) vital signs were subtracted from initial ED values to give individuals’ relative change. Cox regression analysis compared relative hypotension > 7 mmHg with mean time to mortality censored at 30 days. The relative hypotension threshold was refined using a fully adjusted risk tool formed of logistic regression models. Receiver operating characteristics were compared to NEWS2 models with and without incorporation of relative systolic. 5136 (16%) of 32,548 ED attendances were linkable with recent discharge vital signs. Relative hypotension > 7 mmHg was associated with increased 30-day mortality (HR 1.98; 95% CI 1.66–2.35). The adjusted risk tool (AUC: 0.69; sensitivity: 0.61; specificity: 0.68) estimated each 1 mmHg relative hypotension to increase 30-day mortality by 2% (OR 1.02; 95% CI 1.02–1.02). 30-day mortality prediction was marginally better with NEWS2 (AUC: 0.73; sensitivity: 0.59; specificity: 0.78) and NEWS2 + relative systolic (AUC: 0.74; sensitivity: 0.63; specificity: 0.75). Comparison of ED vital signs with recent discharge observations was feasible for 16% individuals. The association of relative hypotension > 7 mmHg with 30-day mortality was externally validated. Indeed, any relative hypotension appeared to increase risk, but model characteristics were poor. These findings are limited to the context of older people with recent hospital admissions.

## Background

Compared to younger people, changes in older people’s vital signs are associated with larger increases in absolute mortality [1]. The most common composite risk score for vital signs, National Early Warning Score v2 (NEWS2), has limitations when applied to older people, having recently been demonstrated to underestimate mortality risk for this group [2, 3]. Absolute thresholds for normal or abnormal continuous data values lack clinical meaningfulness as normal ranges differ in this group, and the overall scores may have less prominence in clinical decision making [4, 5].

**Fig. 1:**
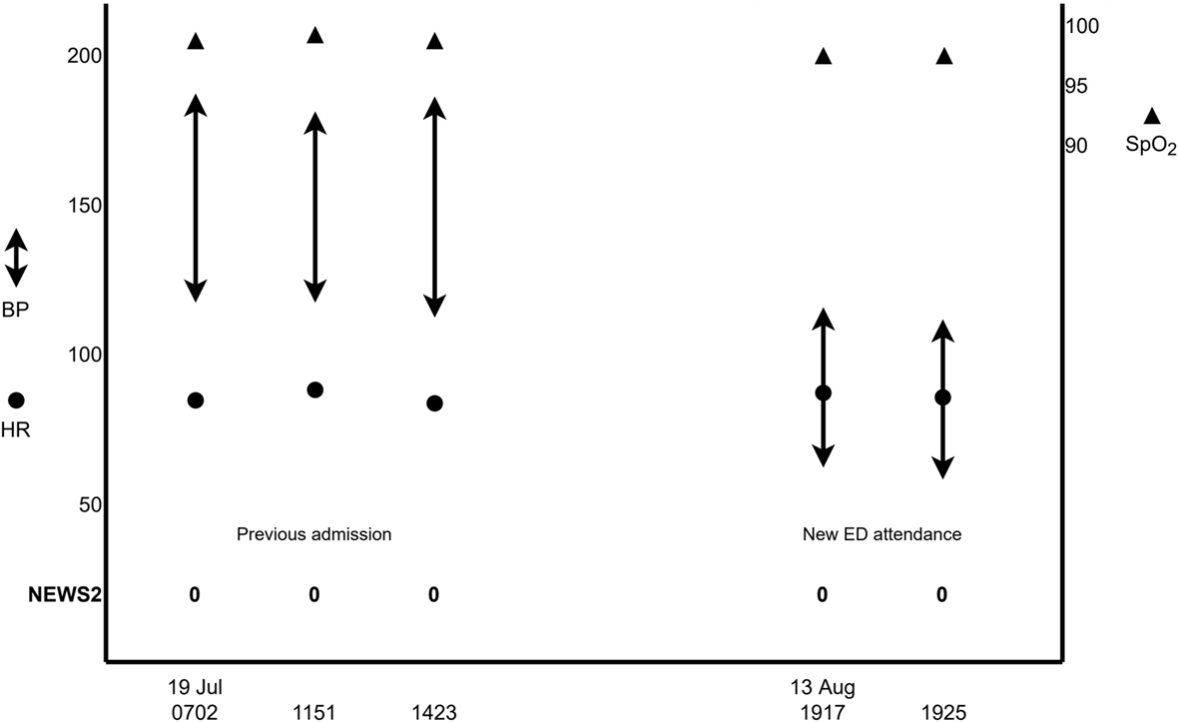
Hypothetical illustration of a person with ‘relative hypotension’ from baseline, unrecognised by normal NEWS2 score.

Age alters physiological responses to acute health problems such as infection and trauma. Older people are more likely to be living with hypertension, and so when NEWS2 is calculated, blood pressure significantly lower than their usual may still fall within the ‘normal’ range (Fig. 1). Similarly, older people more often use medications such as beta blockers which blunt adaptive responses.

There is no established definition for ‘baseline’ blood pressure. A recent Dutch study obtained a value for baseline systolic blood pressure for older people attending Emergency Departments (ED) by querying primary care and outpatient records and taking the mean of three most recent values [6]. Increased mortality was observed in the tercile with greatest relative reduction, among whom systolic pressure was at least 7 mmHg below baseline. However, in practice Emergency Departments often do not have good access to primary care vital signs data, so this may not be a feasible way of establishing baseline blood pressure. Vital signs recorded during previous hospital episodes, however, are often readily available, particularly where systems use electronic patient records (EPR).

It is not known if using previous hospital readings of vital signs can give a useful baseline against which to judge the relative blood pressure change measured on presentation to the ED, or if relative hypotension against this baseline is a risk factor for mortality.

### Objectives

This was a pragmatic initial exploratory analysis using data readily obtainable in real-world emergency care. This study served firstly to assess the feasibility of using previous hospital admission data as a baseline measurement of relative hypotension, and secondly as an external validation of the thresholds derived from primary care data described by Candel et al. [6] which observed greater mortality among people with relative hypotension exceeding 7 mmHg. Finally, we aimed to refine a threshold for relative hypotension that ED clinicians might consider clinically important, developing a risk tool to predict 30-day mortality and comparing the discriminatory performance to NEWS2.

## Methods

This retrospective cohort study analysed data which were electronically collected routinely in the ED and inpatient wards from implementation of an EPR to end-2019. In our UK institution, web-based EPR software (NerveCentre) displays not only current and previous ED vital signs observations, but also those recorded during previous inpatient admissions. The EPR has been used for all adult inpatient observations (except Intensive Care) since 2018. Outpatient and primary care data were not integrated electronically at the time of the study. Our ED is large and is one of the busiest in Europe, with approximately 1000 daily attendances in early-2023. Around one-fifth of attendees are aged over 65 [7].

Available data included demographics, vital signs, and diagnoses as ICD-10 codes. All data were extracted from the EPR using automated export by a data manager who was not involved in study analyses. The observation period was selected to mitigate for a plausible effect on attendance patterns and mortality data during the Covid-19 pandemic. In a deviation from the online protocol, only data for people aged over 64 were analysed due to electronic archiving restrictions at the time of access [8]. Data linkage and analyses were performed using R with caret, comorbidity, ggplot2, HFRS, pROC, and survival packages [9]. The study protocol was approved by the University of Leicester’s research ethics committee (ref 33749) and the UK Health Research Authority (ref 313451). Reporting followed the STROBE guidelines [10].

### Dataset preparation

Data were filtered so that each individual’s first emergency care attendance in year 2019 (excluding Eye Casualty presentations) was taken as their ‘index’ episode. Index episodes were linked to the person’s immediate prior ‘reference’ episode. Eligible reference episodes were hospital admissions which lasted more than seventy-two hours, had admission date within the previous eighteen months (the EPR implementation period) and ended with discharge more than fourteen days before the index attendance.

The mean values for vital signs observed over the final forty-eight hours of reference episodes were considered to represent ‘baseline’ values, as in this period people would have been usually well enough to go home having reached more stable health. Frailty assessments used the Clinical Frailty Score (CFS), and due to data missingness the Hospital Frailty Risk Score (HFRS) was computer-calculated for all patients from ICD-10 codes at reference discharge [11, 12]. The Charlson Comorbidity Index (CCI) was also computer-calculated from ICD-10 codes.

Relative systolic, diastolic, and pulse values were each calculated by subtracting the baseline reference observations from the first index emergency care value. The final dataset therefore linked demographics, vital signs, and outcomes between people’s index ED attendance and immediate prior reference inpatient discharge.

### Statistical analyses

#### Feasibility of comparison with baseline data

The proportion was reported for ED attendances with linkable hospital vital signs data within the previous eighteen months.

#### External validation of the 7 mmHg relative hypotension threshold

Summary statistics were presented for age, frailty, comorbidity, episode characteristics including vital signs, and mortality outcome for emergency care attendees with eligible reference episodes. Cases of relative hypotension were identified as negative relative systolic values exceeding 7 mmHg. 30-day mortality and survival frequencies were subclassified by presence of relative hypotension and clinical variables.

Continuous data for age, HFRS, and vital signs were assessed for normality using visual inspection of density plots and Shapiro–Wilk tests. Age, frailty, and relative hypotension were then examined for interaction using the Kruskal Wallis test. Cox regression analysis assessed for association between relative hypotension (7 mmHg threshold) and mean time to mortality censored at 30 days, adjusting for age, sex, frailty, and comorbidity.

#### Refining the threshold for clinically important relative hypotension and developing a risk tool

Receiver operating characteristic (ROC) curve analysis assessed relative systolic pressure as a predictor of binary 30-day mortality. Area under the curve, sensitivity, and specificity values were determined. The relative hypotension threshold maximising sensitivity and specificity was determined by the Youden Index.

Logistic regression models were used to examine the effect of relative hypotension on binary 30-day mortality, developing a risk tool using forward selection of parameters including age, sex, HFRS, CCI, ethnicity, interaction terms for vital signs and comorbidity, and k-fold internal cross validation (*k* = 10). Next, relative systolic change was added to the NEWS2 model to assess for improved performance. The ROC curves for the new risk tool and for NEWS2 plus relative systolic pressure were compared with NEWS2 alone.

## Results

### Dataset description and feasibility of comparison

**Fig. 2:**
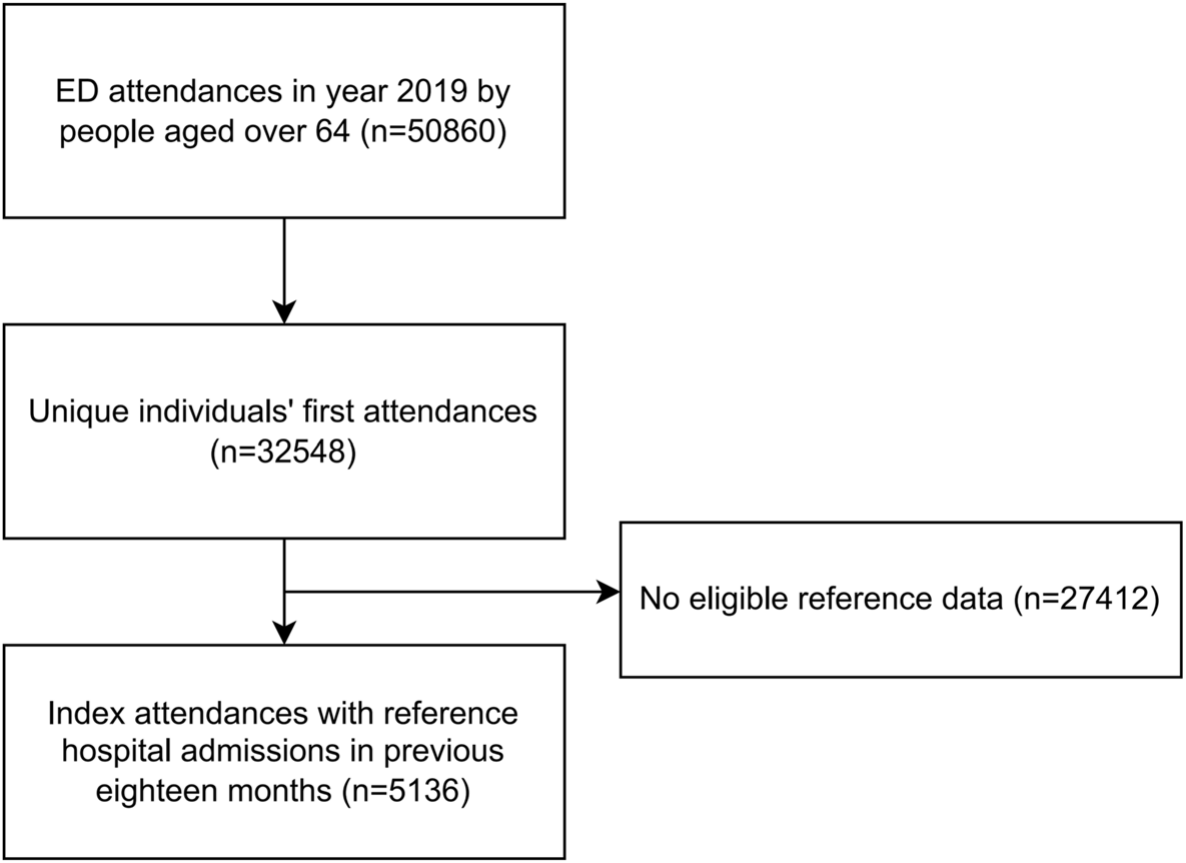
Flow diagram showing inclusion of index attendances with linkable reference data

In 2019 our ED was attended 50,860 times by 32,548 people aged over 64 years, and their first attendances were selected for analyses (Fig. 2). 5136 (16%) index ED attendances had eligible reference hospital episodes for data linkage. These individuals’ characteristics are summarized in Table 1. 3318 (65%) people had died by the time of analysis in 2022 [median 318 (IQR 587) days from ED attendance], and 534 died within 30 days. Nearly half (44.8%) of individuals had missing data for CFS. Where CFS was recorded, those who died within 30 days had higher proportion of frailty (52%) than those who survived (36%). Calculated for the whole cohort, the HFRS was similar between people who died and survived [5.7 (IQR 7.8) vs 5.4 (IQR 8.5), median difference: − 0.4; 95% CI − 0.8 to 0].

**Table 1:** Summary characteristics for individuals included in analyses

Values presented as median (IQR) unless stated	Total cohort *N* = 5136	30-day outcome		Median diff (95% CI)	Odds ratio (95% CI)^d^
		Died *n* = 534	Survived *n* = 4602		
Age, years	81 (13)	83 (13)	81 (13)	− 2 (− 3 to 1)	
Female, *n* (%)	2769 (53.9)	260 (48.7)	2509 (54.5)		0.79 (0.66–0.95)
CFS 5–9^a^, *n* (% of 2833 non-missing)	1929 (68.1)	279 (86.4)	1650 (65.7)		3.30 (2.37–4.70)
CFS missing, *n* (% of cohort)	2303 (44.8)	211 (39.5)	2092 (45.5)		0.78 (0.65–0.94)
HRFS	5.4 (7.8)	5.7 (8.5)	5.4 (7.7)	− 0.4 (− 0.8 to 0)	
CCI	2 (2)	2 (2)	2 (1)	0 (0 to 0)	
Reference interval^b^	225 (256.2)	170.5 (252.8)	231 (252.8)	46 (32 to 60)	
Baseline systolic, mmHg	128.5 (21.4)	126.6 (22.6)	128.8 (21.2)	2.87 (1.42 to 4.33)	
Index systolic, mmHg	137 (36)	123 (36)	138 (35)	15 (12 to 17)	
Index diastolic, mmHg	75 (22)	71 (22.8)	75 (22)	4 (2 to 5)	
Index pulse, min^−1^	80 (25)	88 (30)	80 (23)	− 8 (− 10 to 6)	
Index NEWS2	1 (4)	4 (6)	1 (3)	− 2 (− 3 to 2)	
Δ^c^ systolic, mmHg	7.7 (34.5)	− 2.5 (33)	8.7 (33.9)	11.33 (9 to 13.7)	
Δ diastolic, mmHg	4.6 (20.8)	1.5 (21.9)	4.9 (20.7)	2.7 (1.2 to 4.2)	
Δ pulse, min^−1^	5.2 (20.6)	12.7 (27.7)	4.8 (20)	− 6.75 (− 8.43 to 5.07)	

*CFS* Clinical Frailty Scale, *HFRS* Hospital Frailty Risk Score, *CCI* Charlson Comorbidity Index, *NEWS2* National Early Warning Score v2

^a^CFS produces ordinal data. Percentage of non-missing values reported. CFS 5+  indicates mild or more severe frailty

^b^Interval from reference discharge to index attendance in days

^c^Δ values calculated as index minus reference (baseline) observation

^d^Odds ratios were estimated using Fisher’s Exact Test

#### External validation of the 7 mmHg relative hypotension threshold

People with 30-day mortality had higher frequency of relative hypotension (> 7 mmHg) than those who survived (43.6% vs 26.5%; OR 2.15; 95% CI 1.78–2.59). Frequencies were similar across subclassifications of age, CFS, HFRS, CCI, and index vital signs (Table 2). CFS was omitted from the subsequent analyses due to the extent of missing data; HFRS was used as the frailty measure.

**Table 2:** Subclassification of 30-day mortality by presence of relative hypotension exceeding 7 mmHg threshold

Characteristic	Died within 30 days		Alive after 30 days		Odds ratio (95% CI)^a^
	Relative hypotension > 7 mmHg		Relative hypotension > 7 mmHg		
	*n*	%	*n*	%	
Whole cohort	233	44	1219	26	2.15 (1.78–2.59)
Age					
65–84	132	44	774	26	2.22 (1.73–2.85)
85 +	101	43	445	27	2.04 (1.53–2.73)
Frailty (CFS)					
0–4	20	45	175	20	3.26 (1.66–6.31)
5–6	52	40	308	27	1.85 (1.24–2.73)
7 +	67	45	168	34	1.58 (1.07–2.34)
Frailty (HFRS)					
Low	99	42	548	25	2.11 (1.58–2.80)
Intermed	102	44	558	28	2.05 (1.54–2.73)
High	32	49	113	27	2.66 (1.51–4.70)
Charlson CCI					
0–2	148	43	890	26	2.17 (1.72–2.74)
3–4	73	43	286	28	1.99 (1.40–2.81)
5 +	12	57	43	39	2.03 (0.72–5.98)
Index SBP					
< 111	145	87	495	83	1.38 (0.83–2.39)
111–219	88	24	724	18	1.42 (1.09–1.84)
> 219	NA	NA	NA	NA	NA
Index pulse					
< 51	14	74	17	23	9.11 (2.64–37.20)
51–90	114	40	798	24	2.07 (1.60–2.68)
> 90	105	46	404	32	1.80 (1.34–2.42)
Index NEWS2					
0	18	26	264	15	1.96 (1.06–3.48)
1–4	79	36	661	31	1.29 (0.95–1.74)
5+	136	55	294	41	1.73 (1.28–2.35)

Odds ratios were estimated using Fisher’s Exact test

**Fig. 3:**
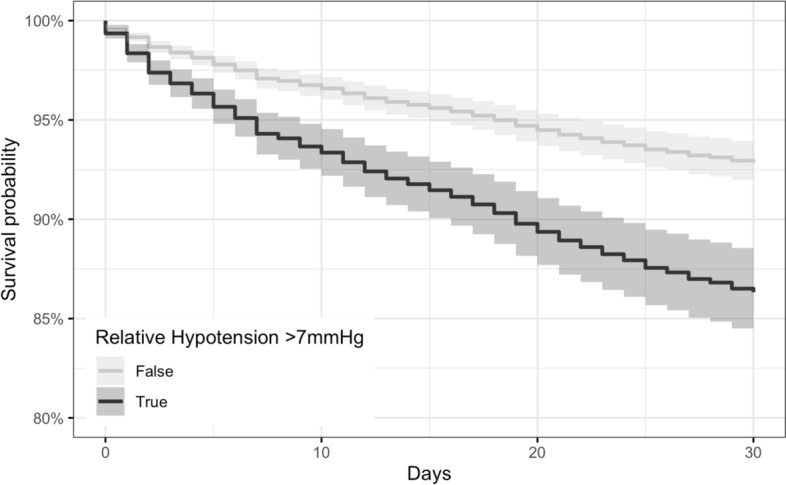
Multivariate Cox regression model for relative hypotension > 7 mmHg and 30-day mortality

In univariate Cox regression models (Table 3), relative hypotension exceeding 7 mmHg below baseline was associated with 107% increased risk (HR 2.07; 95% CI 1.74–2.45) of mortality (censored at 30 days) compared to relative hypotension ≤ 7 mmHg or relative hypertension. This was also the case in the multivariate model, where there appeared to be 98% increased risk (HR 1.98; 95% CI 1.66–2.45) of mortality within 30 days for individuals with relative hypotension exceeding 7 mmHg below baseline, having adjusted for age, HFRS, CCI, and sex. Mortality risk was also higher for people with higher CCI (HR 1.20; 95% CI 1.13–1.28) and male sex (HR 1.23; 95% CI 1.04–1.45), whereas there was no strong association with age (HR 1.03; 95% CI 1.02–1.04) or HFRS (HR 1.00; 95% CI 0.99–1.02). The survival curves for the multivariate Cox model are shown in Fig. 3, demonstrating poorer survival probability for individuals with relative hypotension exceeding 7 mmHg having adjusted for age, HFRS, CCI, and sex.

**Table 3:** Univariate and multivariate Cox regression models for 30-day mortality

	Hazard ratio (95% CI)	
	Univariate models	Multivariate model
Relative hypotension > 7 mmHg	2.07 (1.74–2.45)	1.98 (1.66–2.35)
Age (years)	1.03 (1.02–1.04)	1.03 (1.02–1.04)
HFRS	1.02 (1.00–1.03)	1.00 (0.99–1.02)
CCI	1.22 (1.15–1.30)	1.20 (1.13–1.28)
Sex (male)	1.25 (1.05–1.48)	1.23 (1.04–1.45)

#### Refining the threshold for clinically important relative hypotension and developing a risk tool

A logistic regression model for 30-day mortality and relative systolic pressure (AUC: 0.62; *k*-fold SD_AUC_: 0.03) yielded a surprisingly small 1.81 mmHg (lower than baseline) threshold at the point of maximised sensitivity (0.52; 95% CI 0.48–0.57) and specificity (0.67; 95% CI 0.66–0.68). Using this threshold, 1095 of 4366 individuals with apparently normal ED systolic pressure (> 111 mmHg [13]) had relative hypotension from baseline, and their mortality was higher (11.1% vs 7.5%). These people were very slightly older (mean 81.3 vs 80.8) and had slightly higher HFRS (mean 7.24 vs 6.68). Similarly, among 3372 people with reassuring ED NEWS2 (< 3), 946 had relative hypotension and their mortality was 7.9% vs 4.4%.

**Fig. 4:**
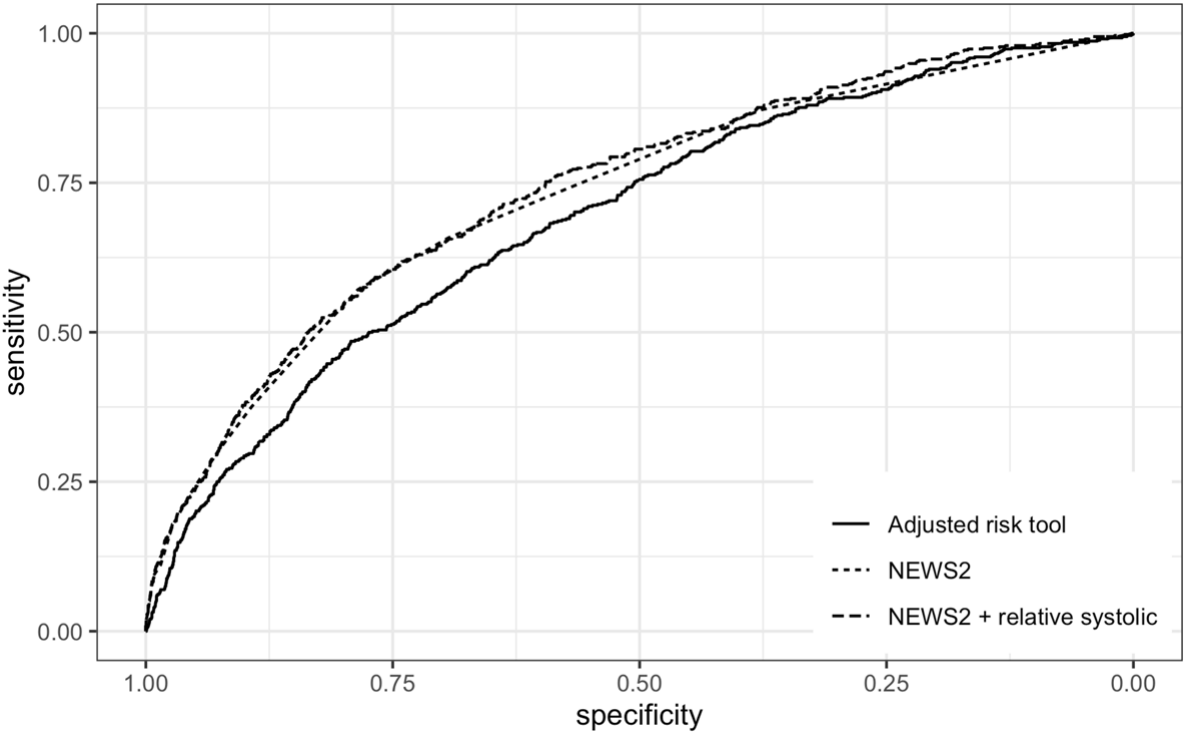
ROC curves demonstrating better performance of NEWS2 with relative systolic pressure compared to NEWS2 alone and the adjusted risk tool

The final risk tool adjusted for all predictors with evidence for significance in univariate models. Greater relative systolic decrease, pulse increase, comorbidity index, age, and male sex were all significant predictors for 30-day mortality (Table 4). AUC for this adjusted risk tool was 0.69 (*k*-fold SD_AUC_: 0.05). The NEWS2 composite score alone had better predictive performance (AUC: 0.73; *k*-fold SD_AUC_: 0.05), while adding relative systolic change to the NEWS2 conferred marginal further improvement (AUC: 0.74; *k*-fold SD_AUC_: 0.03). Model characteristics for the ROC curves (Fig. 4) are presented in Table 5.

**Table 4:** Summary of covariates in the fully adjusted risk tool

	Odds ratio (95% CI)
Relative systolic (1 mmHg decrease)	1.02 (1.02–1.02)
Relative pulse (1 min^−1^ decrease)	0.98 (0.98–0.99)
CCI (from sample mean)	1.23 (1.15–1.30)
HFRS (from sample mean)	1.00 (0.98–1.01)
Age (years from sample mean)	1.03 (1.02–1.04)
Sex (male)	1.23 (1.05–1.44)
Ethnicity	1.02 (0.99–1.04)

**Table 5:** ROC model characteristics

Model	AUC (*k*-fold SD_AUC_)	Sensitivity (95% CI)	Specificity (95% CI)
Adjusted risk tool	0.69 (0.05)	0.61 (0.46–0.78)	0.68 (0.49–0.81)
NEWS2	0.73 (0.05)	0.59 (0.54–0.69)	0.78 (0.69–0.80)
NEWS2 + relative systolic	0.74 (0.03)	0.63 (0.54–0.78)	0.75 (0.58–0.82)

Sensitivity and specificity at Youden’s threshold value

## Limitations

This study used retrospective hospital data from a single centre. Only people aged over 64 were included in the analysis due to archiving, restricting the data accessible for research. The feasibility of comparison with baseline data may be overestimated, with younger people typically having fewer hospital admissions. Furthermore, people with previous hospital admissions are more likely to live with frailty or chronic illness, and therefore interpretation of relative hypotension in primary care or outpatient settings should be with caution. The risk tool presented and the integration of relative systolic with NEWS2 warrant further research with other cohorts.

Data for CFS were missing in approximately half of this sample, despite the scale being in routine use here [14]. Further work is needed to examine the patterns and significance of this missingness. Our analyses therefore instead used the HFRS calculated for all individuals. The CFS considers the phenotypic manifestations of frailty on function and dependence, while the HFRS considers the sensitive diagnoses contributing to frailty-related risks. Elsewhere, these measures have both been shown to predict adverse health outcomes but are only weakly correlated, meaning they may identify different populations who are at risk [15, 16].

Our final model found the Charlson comorbidity index to be associated with 30-day mortality. We did not examine the contributing diagnoses in closer detail, and we did not have access to prescribing data for the included individuals. Investigation of subgroups with specific conditions (such as hypertension) is warranted to better understand this relationship.

We compared models only for the outcome of death within 30 days. Our system lacks routine collection of more person-centred outcomes, which are likely to be more meaningful especially for those living with frailty [17]. Future work might examine relationships between physiological data and person-centred outcomes from healthcare.

## Discussion

Using data routinely available to clinicians, we were able to compare 16% older ED attendees’ vital signs to their recent baseline EPR data for hospital episodes. Systems with more established EPR systems might consider longer reference periods, and as technology evolves it may become possible to ‘pull’ primary care vital signs into the hospital EPR for comparison. Furthermore, such integrations and the increasing use of hospital electronic prescribing may enable future consideration of pharmacological contributors to impaired autoregulation. Our initial analysis provided external validity for increased mortality risk when relative systolic pressure exceeds 7 mmHg below baseline, as observed by Candel et al. previously [6].

To refine this threshold using a risk tool, we considered relative systolic pressure as a continuous variable and observed a surprisingly small 1.81 mmHg (lower than baseline). Test characteristics were poor for sensitivity and specificity, and this threshold is not measurable in practice due to device precision, variation, and calibration. Our data did not report the method for each blood pressure measurement, but we expect the majority to have used automatic cuffs with digital displays. It appeared, then, that in effect any relative hypotension could confer mortality risk.

In our sample, the unaltered NEWS2 had similar performance for 30-day mortality prediction to previous research observing older people living with frailty [3]. Incorporating relative systolic change into a NEWS2 model marginally improved predictive performance for 30-day mortality. This yielded the best-performing AUC in this study, which at only 0.74 prompts for consideration on the limitations of risk stratification scores compared with ED clinician judgement, especially when applied to older people living with frailty [5, 18].

### Theories for the mortality effect of relative hypotension

We had sought to determine whether systolic pressure lower than baseline was associated with mortality, having based our theory on knowledge of hypotension causing poor outcomes. However, re-examination of Table 1 presents an alternative hypothesis. People who died had slightly lower ED systolic pressure than baseline value (mean 123 mmHg vs 127 mmHg), while survivors had relatively higher systolic pressure than their previous baseline (mean 138 mmHg vs 129 mmHg). We postulate that the mechanism underlying our small observed ‘relative hypotension’ threshold was not the relative decrease in these individuals’ systolic pressure, but rather their absence of autoregulatory relative hypertension during illness [19]. Future work might therefore consider risks for people with blood pressure at or below previous discharge values. We assumed that these discharge values represented individuals’ ‘normal’ baseline pressure, but with strict inpatient medications concordance and dynamic physiology during illness recovery this may not have been the case; further work observing hospital discharge and subsequent vital signs in primary care is warranted.

In older people, both high and low blood pressures are associated with poorer outcomes in a range of clinical situations [20–22]. Older people are more likely to experience chronic hypertension, with resulting small vessel disease, arterial stiffness, and reduced arterial compliance [19, 23]. As a result, the brain may become chronically adapted to higher resting perfusion pressures, and may be more vulnerable to acute episodes of hypotension due to a rightward shift in the cerebral autoregulation curve [23, 24]. Cerebral autoregulation is the intrinsic property of the brain to regulate its own blood supply, buffering falls and rises in systemic blood pressure, maintaining oxygen delivery and preventing pressure surges [25]. Therefore, moderately elevated blood pressure during acute illness may confer a protective effect by maintaining consistent cerebral perfusion, and not breaching the lower or upper limits of cerebral autoregulation. However, larger rises in blood pressure may also be detrimental, as with ageing the blood vessels become more fragile, and the risk of cerebral haemorrhage is higher [19].

Observed individuals who survived and died were similar in age and had only slight differences in HFRS and Charlson comorbidity scores. Consistent with previous research, those with higher CFS had poorer 30-day mortality although the degree of missing data was substantial [7]. Further examination for differences between these groups is warranted in order to explain this absence of relative hypertension. In particular, this might consider not only the diagnoses contributing to underlying comorbidity but also individual medications and polypharmacy [26]. The presenting issue and ED diagnosis are also candidates for investigation, with sepsis and trauma plausibly disrupting homeostatic mechanisms for blood pressure compensation [27].

### Conclusions

In this single-centre study, relative change in vital signs was calculated using routine electronic patient records for a modest proportion of ED attendees. Independent external validation was demonstrated for the association between relative systolic decrease exceeding 7 mmHg from baseline and increased mortality within 30 days. An adjusted risk tool in effect showed any relative hypotension to confer increased mortality risk. These findings have restricted context to those older people with previous hospital admissions, and should be interpreted with caution in other settings. Further investigation is needed to identify differences within comorbidity subgroups.

## Data Availability

The dataset generated and analysed during the current study are not publicly available due to the ethical approvals granted but are available from the corresponding author on reasonable request.
